# A word of caution in the functional monitoring of patients after rectal cancer surgery: a multicentre observational study

**DOI:** 10.1007/s10151-024-03089-w

**Published:** 2025-01-04

**Authors:** P. Planellas, N. Fernandes-Montes, T. Golda, S. Alonso-Gonçalves, G. Elorza, J. Gil, E. Kreisler, M. R. Abad-Camacho, L. Cornejo, F. Marinello

**Affiliations:** 1 Colorectal Surgery Unit, University Hospital of Girona, Department of Medical Sciences, Faculty of Medicine University Hospital of Girona, University of Girona, Girona Biomedical Research Institute (IDIBGI), Avinguda de França S/N, 17007 Girona, Spain; 2 Colorectal Surgery Unit, Vall d’Hebron University Hospital, Universitat Autònoma de Barcelona UAB, Barcelona, Spain; 3 Colorectal Surgery Unit, Bellvitge University Hospital, University of Barcelona, IDIBELL (Bellvitge Biomedical Investigation Institute), Barcelona, Spain; 4 Colorectal Surgery Unit, Hospital del Mar; Medical Research Institute (IMIM), Barcelona, Spain; 5 Colorectal Surgery Unit, University Hospital of Donostia, Donostia, Spain; 6 Colorectal Surgery Unit, Hospital Universitario de Gran Canaria Dr. Negrín, Gran Canaria, Spain; 7 Girona Biomedical Research Institute (IDIBGI), Girona, Spain

**Keywords:** Rectal cancer, Functional follow-up, Surgical sequelae, Monitoring

## Abstract

**Background:**

Patients with rectal cancer often experience adverse effects on urinary, sexual, and digestive functions. Despite recognised impacts and available treatments, they are not fully integrated into follow-up protocols, thereby hindering appropriate interventions. The aim of the study was to discern the activities conducted in our routine clinical practice outside of clinical trials.

**Methods:**

This multicentre, retrospective cohort study included consecutive patients undergoing rectal cancer surgery between January 2016 and January 2020 at six tertiary Spanish hospitals.

**Results:**

A total of 787 patients were included. Two years post surgery, gastrointestinal evaluation was performed in 86% of patients. However, bowel movements per day were only recorded in 242 patients (46.4%), and the values of the Low Anterior Resection Syndrome (LARS) questionnaire were recorded in 106 patients (20.3%); 146 patients received a diagnosis of fecal incontinence (28.2%), while 124 patients were diagnosed with low anterior resection syndrome (23.8%). Urogenital evaluation was recorded in 21.1% of patients. Thirty-seven patients (5.1%) were detected to have urinary dysfunction, while 40 patients (5.5%) were detected to have sexual dysfunction. A total of 320 patients (43.9%) had their quality of life evaluated 2 years after surgery, and only 0.8% completed the Quality of Life questionnaire. Medication was the most used treatment for sequelae (26.9%) followed by referral to other specialists (15.1%).

**Conclusions:**

There is a significant deficit in clinical follow-ups regarding the functional assessment of patients undergoing rectal cancer surgery. It is crucial to implement a postoperative functional follow-up protocol and to utilize technologies such as Patient-Reported Outcome Measures (PROMs) to enhance the evaluation and treatment of these sequelae, thereby ensuring an improved quality of life for patients.

**Supplementary Information:**

The online version contains supplementary material available at 10.1007/s10151-024-03089-w.

## Introduction

Patients diagnosed with rectal cancer will likely experience a reduced quality of life, affecting urinary, sexual, and digestive functions [1–3]. Despite the widespread recognition of these effects and the availability of treatments aimed at their correction or at least mitigation, they have not yet been fully incorporated into patient follow-up protocols [4, 5]. The absence of a consensus on how to detect and address these effects currently impedes the implementation of appropriate interventions to meet patients’ needs [6, 7]. Although there is growing acknowledgement of the importance of patient-recorded outcomes and quality of life, until now research in rectal cancer has predominantly focused on assessing local or distant control and overall survival [8].

In routine clinical practice, beyond clinical trials that employ complex scales to evaluate surgical sequelae and changes in quality of life, strict monitoring of these domains is rare. Despite having various oncological follow-up protocols for rectal cancer [9, 10], there are currently no guidelines for detecting alterations induced by surgical or radiochemotherapeutic treatments. Consequently, we are at risk of encountering the “symptom iceberg” [11]—only identifying a fraction of patients (the visible surface of the iceberg) and failing to fully recognise patients’ sequelae derived from treatment.

Functional alteration and quality of life outcomes exhibit variation dependent on a multitude of factors as indicated by several studies; for example, lower tumour lesions, advanced patient age, male gender, obesity, a narrow pelvis, and the utilisation of neoadjuvant treatment have all been correlated with heightened surgical complexity, suboptimal oncological outcomes, and poorer functional results [12, 13]. These diverse variables underscore the complexity of the interplay between patient characteristics and treatment approaches, highlighting the need for a nuanced and individualised approach to patient care in the context of cancer management [14]. The Hawthorne effect is observed in physicians participating in studies, because their actions differ from those in routine clinical practice [15].

With this work, we aim to discern the activities conducted in our routine clinical practice, i.e., outside of clinical trials, and evaluate the quality of functional follow-up and the quality of life in patients with rectal cancer over the 2 years following surgery.

## Methods

### Study design

This study was designed as a retrospective, multicentre, observational study conducted at six tertiary Spanish hospitals with specialised colorectal surgery units. The protocol was approved by the ethical committees of participating hospitals (No. 2022.046).

### Patient selection

We identified patients who underwent consecutive curative-intent elective resection for rectal cancer between January 2016 and January 2020.

The inclusion criteria were as follows: elective operation for rectal cancer with an attempt at R0 resection, histologically confirmed adenocarcinoma located within 15 cm from the anal margin, aged 18 years or older, any T, N, or M stage, with or without neoadjuvant therapy. Exclusion criteria included patients who died during the first postoperative year and patients who were enrolled in functional clinical trials (see Supplementary material).

Patients with temporary or permanent stoma at different time points were excluded from the gastrointestinal evaluation.

### Patient follow-up

We extracted follow-up data from medical records and analysed it over the course of the first and second years after surgery. This data encompasses functional and quality of life assessment.

For assessing gastrointestinal function, we analysed recorded clinical notes of fecal incontinence during patients’ follow-up. This involved documenting bowel movement frequency, recording episodes of fecal or gas incontinence, and either utilising the LARS score [16] or other defecatory function assessment scales, such as St. Mark’s incontinence score [17] or Wexner Continence score [18].

To assess urinary and sexual function, we evaluated the recorded clinical information based on patients’ genitourinary functionality, and determined whether scales, such as the International Consultation on Incontinence Questionnaire-Short Form (ICIQ) [19], the Female Sexual Functional Index (FSFI) [20], or the International Index of Erectile Function (IIEF-5) [21], were used to evaluate patient’s genitourinary condition.

Regarding quality of life, we recorded whether the clinical notes included recorded information about patients’ physical and emotional status, and whether a questionnaire, such as SF-36 [22], was used to measure the impact on the quality of life.

Additionally, we investigated whether any interventions were implemented to address patients’ sequelae. These interventions include pharmacological treatments, referrals to specialists, or targeted therapies such as transanal irrigations, sacral neuromodulation, and posterior tibial neuromodulation.

### Outcome

The aim of this study was to discern the activities performed in our routine clinical practice outside of clinical trials. We evaluated the quality of functional follow-up and the quality of life among patients with rectal cancer over the 2 years following surgery, with the intention of identifying potential areas for improvement and developing standardized follow-up protocols.

### Statistical methods

Descriptive analyses were conducted of demographic, clinical, surgical, and postoperative variables together with the functional assessment at 1 and 2 years post surgery. Variables are presented as median and interquartile range (IQR) for continuous data and frequencies and percentages for categorical variables. We used SPSS v. 20.0 (SPSS Inc, Chicago, IL, USA).

## Results

### Patients’ characteristics

A total of 787 patients were included in the study; 534 (67.9%) men and 253 (32.1%) women were included in the analyses (see Supplementary material). Table 1 reports the characteristics of these patients. Median patient age was 67 years (IQR 59–75), body mass index (BMI) was 26.5 (IQR 23.5–29.1), and 413 (52.5%) were classified as American Society of Anesthesiologists (ASA) III or IV. Rectal cancer was classified as stage T3 in 456 patients (57.9%) and as stage N1 or N2 in 467 patients (59.3%). Tumour height from the anal verge was 10–15 cm in 257 patients (32.7%), 5–10 cm in 334 (42.4%), and less than 5 cm in 176 (22.4%); 76 (9.7%) had synchronic metastases. Neoadjuvant therapy was administered in 471 patients (59.8%).

**Table 1:** Patients demographic, clinical, and surgical characteristics

	*N* = 787	%
Female (%)	253	32.1
Age [median (IQR)]	67 (59–75)	
BMI [median (IQR)]	26.5 (23.5–29.1)	
ASA score (%)		
I	22	2.8
II	343	43.6
III	396	50.3
IV	17	2.2
Missing data	9	1.1
Height from anal verge (cm) [median (range)]	8.6 (5.7–12)	
Height from anal verge (cm)		
Low (0–5)	176	22.4
Mid (5–10)	334	42.4
High (10–15)	257	32.7
Missing data	20	2.5
cT		
T1	16	2.0
T2	149	18.9
T3	456	57.9
T4	112	14.2
TX	54	6.9
cN		
N0	237	30.1
N+	467	59.3
NX	83	10.5
Synchronous metastases	76	9.7
Neoadjuvant treatment	471	59.8
Operative procedure		
Exenteration	6	0.8
APR	126	16.0
LAR + anastomosis	611	77.6
LAR + colostomy	42	5.3
Total proctocolectomy	2	0.3
Anastomosis (if applicable)		
Handsewn	67	10.9
Stapled	548	89.3
Missing data	2	0.3
Approach		
Open	48	6.1
Laparoscopy	501	63.7
Robot	155	19.7
TaTME	83	10.5
Conversion (if applicable)		
Yes	78	10.6
No	661	89.4
Stoma		
Defunctioning ileostomy	357	45.4
Defunctioning colostomy	1	0.1
End colostomy	169	21.5
No stoma	260	33.0

*APR* abdominoperineal resection,* ASA* American Society of Anesthesiologists, *IQR* Interquartilie range, *BMI* body mass index, *LAR* low anterior resection, *TaTME* transanal total mesorectal excision

### Surgical variables and postoperative outcomes

Rectal anterior resection was performed in 653 patients (82.9%), whereas abdominoperineal amputation was performed in 126 patients (16.0%). The remaining eight surgeries were six exenterations and two proctocolectomies. Anastomoses were handsewn in 67 patients (10.9%) and stapled in 548 cases (89.3%). Laparoscopy was the main surgical approach (63.7%), followed by robotic, TaTME, and laparotomy. Conversion rate was 10.6%.

Protective ileostomy was performed in 357 patients (45.4%), protective colostomy in 1 patient (0.1%), end colostomy in 169 patients (21.5%), and 260 patients were stoma-free (33%).

As shown in Table 2, 311 patients (39.5%) had early postoperative complications. Minor complications (< IIIa) were identified in 207 patients (26.3%), whereas 104 additional patients (13.2%) had major complications (≥ IIIa). Anastomotic leak was detected in 54 patients (8.8%). Eighty-five patients underwent a re-intervention (10.8%) and 81 patients were readmitted (10.3%). The median of postoperative stay was 7 (IQR 5–12) days.

**Table 2:** Patients’ postoperative and tumour characteristics

	*N* = 810	%
Overall complication	322	39.8
Clavien–Dindo classification		
No complication	488	60.2
I	73	9.0
II	141	17.4
IIIa	14	1.7
IIIb	73	9.0
IVa	20	2.5
IVb	1	0.1
Re-admission	81	10.0
Re-intervention	88	10.9
Anastomotic leakage	56	8.8
Hospital stay (days) [median (IQR)]	7 (5–12)	

### Clinical care assessment 1 and 2 years post surgery

**Fig. 1:**
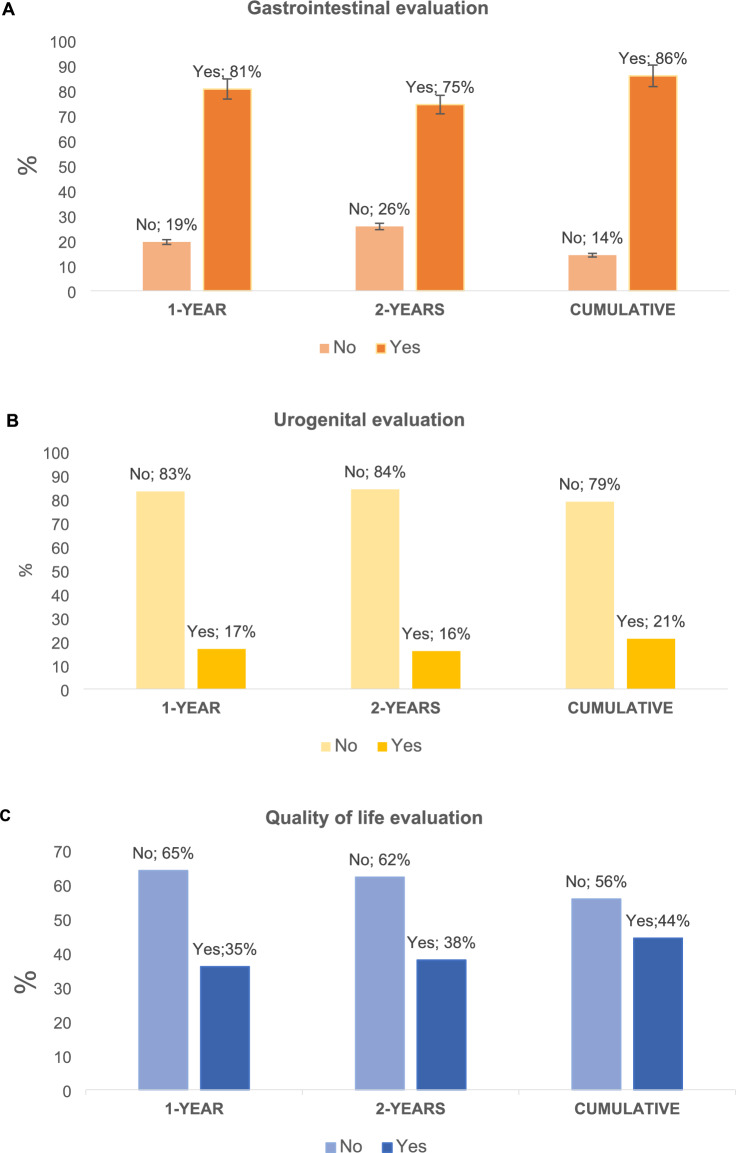
Clinical care assessment at first and second year of follow-up and cumulative rates 2 years post surgery

Table 3 reports the clinical assessment performed by surgeons during the first and second years after surgery, and Fig. 1 shows rates according to gastrointestinal, urogenital, and quality of life evaluations. Follow-up was performed in 772 patients during the first year (15 patients were lost during follow-up) and in 729 patients during the second year (32 patients were lost during follow-up and 26 died after the first year surgery).

**Table 3:** Gastrointestinal and urogenital function evaluation at 1 and 2 years post rectal cancer surgery

		1 year post surgery		2 years post surgery		Cumulative	
		*N* = 772	%	*N* = 729	%	*N* = 729	%
Gastrointestinal evaluation							
Yes		361	80.6	385	74.5	449	86.0
Number of depositions/day recorded							
Not recorded		275	58.8	345	66.7	280	53.6
Recorded		173	37.0	172	33.3	242	46.4
Fecal incontinence assessment							
No fecal incontinence		265	59.2	269	52.0	292	56.5
Fecal incontinence		90	20.1	106	20.5	146	28.2
Not recorded		93	20.8	142	27.5	79	15.3
LARS assessment							
No LARS		280	62.5	288	55.7	319	61.1
LARS		79	17.6	92	17.8	124	23.8
Not recorded		89	19.9	137	26.5	79	15.1
LARS score recorded							
No		377	84.2	446	86.3	416	79.7
Yes		71	15.8	71	13.7	106	20.3
Urogenital evaluation							
Yes		130	16.8	116	15.9	154	21.1
Sexual function assessment							
No dysfunction		47	6.1	48	6.6	61	8.4
Dysfunction		27	3.5	27	3.7	40	5.5
Not recorded		698	90.4	654	89.7	628	86.1
Use of sexual dysfunction scale							
No		770	99.7	728	99.9	726	99.6
Yes		2	0.3	1	0.1	3	0.4
Urinary function assessment							
No dysfunction		102	13.2	89	12.2	112	15.4
Dysfunction		25	3.2	23	3.2	37	5.1
Not recorded		644	83.4	615	84.6	578	79.5
		1	–	2	–	2	–
Use of urinary dysfunction scale							
No		767	99.4	726	99.6	722	99.0
Yes		5	0.6	3	0.4	7	1.0
Quality of life evaluation							
Yes		273	35.4	274	37.6	320	43.9
Use of quality of life scale							
No		766	99.2	729	100.0	723	99.2
Yes		6	0.8	0	0.0	6	0.8
Prescribed treatments							
Neuromodulator		9	1.2	21	2.9	25	3.4
Medication		144	18.7	132	18.1	196	26.9
Nutritionist		57	7.4	25	3.4	65	8.9
Rehabilitator		40	5.2	47	6.4	68	9.3
Urologist		28	3.6	20	2.7	32	4.4
Psychologist		13	1.7	7	1.0	13	1.8
Other treatments		10	1.3	20	2.7	25	3.4

One year after rectal cancer surgery, bowel movements per day were recorded in the medical notes of 37% of patients, fecal incontinence in 90 patients (20.1%), and LARS in 79 patients (17.6%). However, only 15.8% of patients had their LARS score measured.

Sexual and urinary function were missing in the medical notes for more than 90% and 83% of surgical patients, respectively. The use of questionnaires to assess urogenital function after surgery was 0.3% for sexual function and 0.6% for urinary function.

The impact of surgery on patients’ quality of life was recorded in only 273 patients (35.4%) and quantified through the questionnaire in just 0.8% of patients.

Prescribed treatments in patients who have experienced digestive and/or genitourinary sequelae 1 year post surgery were as follows: sacral neuromodulation, *n* = 9 (1.2%); rehabilitation, *n* = 40 (5.2%); medication, *n* = 144 (18.7%); and/or referral to other specialists, *n* = 98 (12.7%) and other treatments, *n* = 10 (1.3%) which include end colostomy formation, transanal irrigations, and hyperbaric chamber.

In the second year of follow-up, 172 patients (33.3%) had their bowel movements recorded in the medical notes. LARS and fecal incontinence after 2 years were recorded in 17.8% and 20.5% of patients, respectively. The LARS score was measured in 71 patients (13.7%).

Sexual function was recorded in 10.3% of patients and urinary function in 15.4%. Questionnaires to quantify urinary and sexual function after 2 years were administered to 0.4% (3 patients) and 0.1% (1 patient), respectively. There was no use of questionnaires to assess emotional and physical well-being at 2 years after surgery.

Patients who developed genitourinary and/or digestive sequelae 2 years following surgery were prescribed the following treatments: sacral neuromodulation (*n* = 21, 2.9%), rehabilitation (*n* = 47, 6.4%), medication (*n* = 132, 18.1%), referral to other specialists (*n* = 52, 7.1%), and other treatments (*n* = 20, 2.7%), including those mentioned previously.

At the 2-year follow-up, the number of bowel movements per day was recorded in 242 patients (46.4%). Fecal incontinence was experienced by 146 patients (28.2%), and LARS by 124 patients (23.8%). The LARS questionnaire was completed by 106 patients (20.3%) at 2 years after surgery.

Urinary dysfunction was recorded in 5.1% of surgical patients, and sexual dysfunction in 5.5%. However, sexual and urogenital function were not recorded in the medical notes for over 80% and 70% of patients, respectively.

Only 320 patients (43.9%) had their quality of life after surgery evaluated, and just 0.8% of patients completed a Quality of Life questionnaire.

Sacral neuromodulation (*n* = 25; 3.4%), rehabilitation (*n* = 68; 9.3%), medication (*n* = 196; 26.9%), and/or referral to other specialists (*n* = 110; 15.1%) and other treatments (*n* = 25; 3.4%), including end colostomy formation, transanal irrigations, and hyperbaric chamber, were prescribed for patients who experienced digestive and/or genitourinary sequelae 2 years post surgery.

## Discussion

Our findings are a warning signal regarding the lack of data collection on functionality and quality of life of our patients during the initial 2 years following rectal cancer surgery. This study involved the participation of six tertiary centres dedicated to treating rectal cancer, which have a relevant trajectory in this field [23, 24]. The results suggest the presence of the Hawthorne effect among the professionals involved, as we have observed a lower detection of alterations in quality of life and urinary, sexual, and defecatory functions than expected. Despite having recorded a large amount of data and having employed complex scales in studies, in routine clinical practice, many of these data are not recorded, and the current scales, which tend to be long and tedious, are underutilised.

During this timeframe, only 43.9% of physicians assessed patients’ quality of life, and a mere 0.8% utilised questionnaires for this purpose. Additionally, 13.9% documented sexual function, while 20.5% recorded urinary function. Questionnaires were used by 1% to assess urinary function and by 0.4% to evaluate sexual function. Among patients without a stoma, only 46.4% recorded daily bowel movement counts, with 23.8% showing signs of LARS and 28.2% reporting fecal incontinence. The rate of treatment for functional sequelae at 2 years post surgery has significantly fallen below expectations. Medication has been prescribed to treat functional sequelae in merely 26.9% of patients. Furthermore, 9.3% of patients have been referred to rehabilitation, while 15.1% have been sent to see other specialists, including urologists, nutritionists, and psychologists, distinct from rehabilitation. The rate of sacral neuromodulation testing 2 years post anterior rectal resection in patients without stoma stands at 3.4%.

Although the literature reports an incidence of alteration in defecatory function after rectal cancer surgery, measured by the LARS scale, of up to 90%, with the prevalence of severe LARS even exceeding 60% [16]. Moreover, even in patients under organ preservation protocols (watch-and-wait approach) there was a 33.3% prevalence of LARS at 24 months [25]. In our series, the specific rate of digestive function assessment 2 years post surgery was 86%. However, the use of the LARS scale outside of clinical trials was only 20.3%.

Urinary dysfunction was recorded in 20% of patients, and sexual dysfunction in 29.5% 1 year post surgery according to previous studies [19, 20], with more problems observed in survivors with a permanent ostomy [26]. In our series, urinary dysfunction was identified in 5.1% of patients, and sexual dysfunction in 5.5% 2 years post surgery. This discrepancy arises from inadequate documentation of sexual and urinary functionality assessments, as sexual function was only recorded in 13.9% of patients and urinary function in 20.5% of patients. These data imply that there is a lack of rigorous monitoring of functional sequelae.

Examining functional outcomes post rectal cancer surgery is essential for a comprehensive understanding, despite its inherent complexity [23]. Clinical trials focused on functional follow-up provide crucial insights into prevalent sequelae, their timing, severity, and effective treatment approaches. Despite the availability of sufficient tools to measure functional outcomes in patients who have undergone rectal cancer surgery, several factors can limit both the asking of questions about functionality during follow-up and the quantitative assessment of it. These factors include the lack of time during consultations, clinical priorities focused on the treatment and monitoring of the oncological disease, limited resources at centres, and the additional burden that questionnaires represent. To improve the quality of care and ensure that patients can actively participate in their own recovery process, it is crucial to have open discussions and allow patients to express themselves [27].

Simplifying strategies applicable in routine clinical practice is equally vital, and technology can play a key role in this regard. Modern tools such as Patient-Recorded Outcome Measures (PROMs) can streamline the identification of affected patients, allowing for a more in-depth assessment [21].

Furthermore, other potential associated causes that could contribute to this lack of rigorous monitoring of functional sequelae in patient follow-up may include a shortage of time during consultations, insufficient expertise among many surgeons in functional sequelae, a lack of active symptom exploration, and a lack of awareness regarding therapeutic possibilities in addressing these sequelae [28–30].

This study cannot be conceived as a clinical audit because of the lack of quality standards with which to compare. We believe that this is a research avenue that scientific societies should explore. After analysing our results, we will implement a postoperative functional follow-up protocol beyond oncological monitoring for the early detection of recurrence in rectal cancer. The goal will be to provide a greater opportunity to identify patients who may require some treatment to improve the quality of life and mitigate functional sequelae throughout their recovery process [31].

## Limitations

This study has limitations such as its retrospective and non-randomised design carried out across six different specialised centres for rectal cancer. Additionally, it encompasses the period of the COVID-19 pandemic, which could have impeded patient follow-up [32, 33].

The absence of templates or protocols for systematic quality of life and functionality assessment complicates the interpretation of follow-up notes. Recognising these constraints emphasises the need for structured approaches in evaluating these critical aspects. Despite these limitations, this review serves as a call to action, prompting initiatives to address this deficit. The study’s insights are expected to provide valuable contributions to daily clinical practice. Acknowledging these limitations presents an opportunity for refining our approach to patient care and assessing treatment outcomes in rectal cancer.

## Conclusions

There is a significant deficit in clinical follow-ups regarding the functional assessment of patients undergoing rectal cancer surgery. The absence of systematic monitoring for alterations in quality of life and disturbances in urinary, sexual, or digestive functions outside of clinical trial settings is identified.

The recommendation to incorporate functionalism into follow-up protocols for patients with rectal cancer aims to achieve a comprehensive, patient-centred change, surpassing current limitations, and recognizing challenges beyond the oncological outcomes of the disease.

## Supplementary Information

Below is the link to the electronic supplementary material.

Supplementary file1 (DOCX 20 KB)

## Data Availability

No datasets were generated or analysed during the current study.
