# Supplementary material for: A word of caution in the functional monitoring of patients after rectal cancer surgery: a multicentre observational study
Source: Tech Coloproctol. 2025 Jan 4;29(1):42. doi: 10.1007/s10151-024-03089-w (PMC11700041; doi:10.1007/s10151-024-03089-w)
Supplement: Supplementary file 1 — Supplementary file1 (DOCX 20 KB) [file 10151_2024_3089_MOESM1_ESM.docx]

**Figure Suppl**. Flowchart of the study

Patients undergoing elective surgery for rectal adenocarcinoma between January 2016 and January 2020

n=837

**Excluded n=50**

Patients who died during the first postoperative year n=27

Patients who were enrolled in functional clinical trials n=23

Patients included in the study

n=787
